# Screening rules for growth to detect celiac disease: A case-control simulation study

**DOI:** 10.1186/1471-2431-8-35

**Published:** 2008-09-11

**Authors:** Paula van Dommelen, Floor K Grote, Wilma Oostdijk, Sabine MPF de Muinck Keizer-Schrama, Bart Boersma, Gerard M Damen, Cassandra G Csizmadia, Paul H Verkerk, Jan M Wit, Stef van Buuren

**Affiliations:** 1 Dept. of Statistics, TNO Quality of life, Leiden, The Netherlands; 2 Dept. of Pediatrics, Leiden University Medical Center, Leiden, The Netherlands; 3 Dept. of Pediatrics, Erasmus MC – Sophia Children's Hospital, Rotterdam, The Netherlands; 4 Dept. of Pediatrics, Medical Center Alkmaar, Alkmaar, The Netherlands; 5 Dept. of Pediatrics, Radboud University Medical Center, Nijmegen, The Netherlands; 6 Dept. of Child Health, TNO Quality of life, Leiden, The Netherlands; 7 Dept. of Methodology & Statistics, University of Utrecht, The Netherlands

## Abstract

**Background:**

It is generally assumed that most patients with celiac disease (CD) have a slowed growth in terms of length (or height) and weight. However, the effectiveness of slowed growth as a tool for identifying children with CD is unknown. Our aim is to study the diagnostic efficiency of several growth criteria used to detect CD children.

**Methods:**

A case-control simulation study was carried out. Longitudinal length and weight measurements from birth to 2.5 years of age were used from three groups of CD patients (n = 134) (one group diagnosed by screening, two groups with clinical manifestations), and a reference group obtained from the Social Medical Survey of Children Attending Child Health Clinics (SMOCC) cohort (n = 2,151) in The Netherlands. The main outcome measures were sensitivity, specificity and positive predictive value (PPV) for each criterion.

**Results:**

Body mass index (BMI) performed best for the groups with clinical manifestations. Thirty percent of the CD children with clinical manifestations and two percent of the reference children had a BMI Standard Deviation Score (SDS) less than -1.5 and a decrease in BMI SDS of at least -2.5 (PPV = 0.85%). The growth criteria did not discriminate between the screened CD group and the reference group.

**Conclusion:**

For the CD children with clinical manifestations, the most sensitive growth parameter is a decrease in BMI SDS. BMI is a better predictor than weight, and much better than length or height. Toddlers with CD detected by screening grow normally at this stage of the disease.

## Background

One of the goals of growth monitoring in developed countries is the detection of undiagnosed illnesses. Nevertheless, there is little consensus on which referral criteria for children with growth retardation are appropriate [1]. Recently we reported on the predictive value of various growth criteria for the detection of Turner's syndrome [2]. The focus of that study was on short stature and slowed growth for length or height, as short stature is the main common physical characteristic of Turner's syndrome. Growth retardation, however, may also imply failure to thrive in terms of slowed growth for weight and BMI.

Celiac disease (CD), also known as gluten-sensitive enteropathy, is characterized by subtotal villous atrophy of the small intestine, intra-epithelial lymphocytosis and crypt hyperplasia, and is associated with a variable mode of presentation. The classical presentation is characterized by failure to thrive, diarrhoea, irritability, vomiting, anorexia, foul stools, abdominal distension and muscle wasting. However, many infants, toddlers and children with celiac disease present with few or no signs and symptoms [3-6]. The prevalence of the classical presentation of CD decreased in the past decade, while the prevalence of non-classical presentations increased [3-5]. Growth failure in terms of length (or height) or weight may be the earliest sign of the disease [7]. In 1994, the reported incidence of clinically diagnosed CD in the Netherlands was 0.54 per 1000 live births [8]. However, screening studies using detection of anti-endomysium antibodies have shown a much higher prevalence (1:300 to 1:100). The ratio of clinically diagnosed versus CD detected by screening varies between 1:7 and 1:14 [9]. Early detection and treatment with a gluten-free diet is required to improve the immediate quality of life of the CD patients and to decrease the long-term risks, including reduction in adult height, a higher prevalence of malignancies, adverse pregnancy outcome, neurological problems and osteomalacia [10].

Mass screening for CD using specific antibodies is unlikely to be performed, because of the uncertainty concerning the cost-benefit ratio. As there is a high incidence of CD (1.7 to 8.3%) in children with growth retardation without gastrointestinal symptoms and even higher (up to 59.1%) when other (endocrine) causes for short stature are excluded [7], a substantial proportion of infants and children with CD may be detected through growth monitoring.

In the Netherlands, nearly every child is monitored for height and weight from birth till the age of 16–18 years. Children with abnormal growth are referred to secondary health care providers according to certain criteria [11]. As no pathologic causes for short stature were detected in most of these referred children, we recently revised the referral criteria. These criteria minimize the unnecessary referrals and are aimed at not missing important diseases such as CD, Turner's syndrome and endocrine abnormalities [12].

So far, it is generally assumed that most CD patients have a slowed growth in terms of length (or height) and weight [13]. However, the effectiveness of slowed growth as a tool for identifying children with CD is unknown. The aim of this study is to establish optimal referral criteria based on abnormal growth for detecting asymptomatic and symptomatic children with CD.

## Methods

### Patients

**Figure 1:**
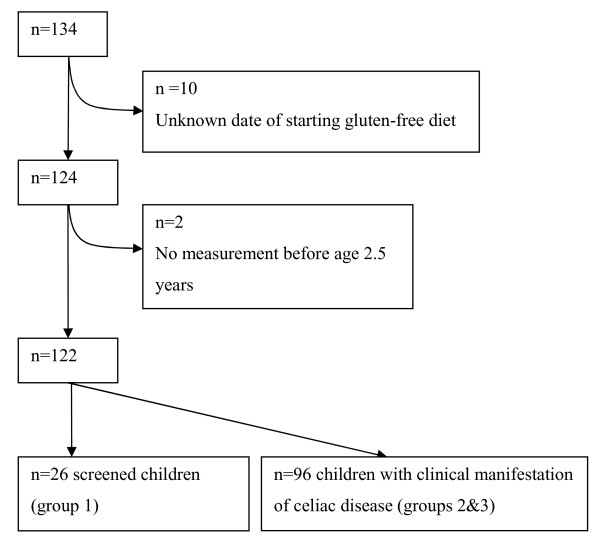
Flow chart of children with CD used in the study.

Longitudinal length and weight data of patients with CD were collected from three different studies. The first study was a prospective screening study using blood tests in unrecognized CD in children aged 2–4 years, visiting the Community Child Health Care Centers in the Dutch province of Zuid (South)-Holland [9]. In this study, 32 children with CD were detected between May 1997 and June 1998. The second study was a retrospective study on catch up growth in patients with CD [13]. A written questionnaire including their symptomatology, duration of complaints before diagnosis, age at diagnosis, associated diseases in the past and parental heights was sent to all members of the Dutch Celiac Society in the early nineteen eighties. Growth data were collected from 74 children younger than 16 years. The third study was a prospective study on catch up growth [14]. All newly diagnosed childhood CD patients from two separate pediatric departments were included between April 1994 and September 1995 (n = 28). The children in the second and third study presented with a full range of classical symptoms. We used all growth data before and at the start of the gluten-free diet, till the age of 2.5 years. The data was gathered retrospectively from child welfare clinics, pediatricians and general practitioners. Additional growth information of these children was obtained from physicians in the Regional Child Health Care Centres. The diagnosis of CD was confirmed by histology for all patients, although in the retrospective study we were dependent on the information provided by patient reports. In total, we included 134 children: 32 children from the first study, 74 children from the second study and 28 children from the third study. Exclusion criteria were: an unknown date of starting the gluten-free diet and no measurement between birth and 2.5 years of age. After excluding such cases, 122 children were eligible for further analyses: 26 children from the first study and 96 children from the second and third study (see Figure 1).

The first CD group was asymptomatic or featured symptoms that were not signalled by the parents or the general practitioners. Therefore, this group was analyzed separately (screened group). The second and third CD groups were clinically diagnosed and we reasoned that these two groups could be pooled (symptomatic group).

### Reference sample

A reference sample was obtained from the Social Medical Survey of Children Attending Child Health Clinics (SMOCC) cohort, a nationally representative cohort of 2,151 children born in the Netherlands during 1988–1989 [15]. Of this cohort longitudinal data of length and weight of children from birth to 2.5 years of age were available. The length and weight from birth to two years of these children were previously described by Herngreen et al.[16].

### Power analysis

For an estimated sensitivity of 50% we obtained a 95% confidence interval (95%-C.I.) of +/- 19% with the 26 screened CD children and +/- 10% with the 96 symptomatic CD children. For an estimated specificity of 98% we obtained a 95%-C.I. of +/- 0.6% with the 2,151 reference children.

### Screening rules

We formulated several screening rules for growth that could serve as criteria for referral to specialist care (Table 1). Each of these rules combines several parameters, such as starting age or a decrease in standard deviation score (SDS) over a certain time period. Table 1 explains the interpretation of each parameter. We used several simulation values for each parameter to see how the diagnostic performance of each rule changes. For example, for the parameter starting age, we used simulation values of 0, 1/2 and 1 year of age. These simulation values were chosen to investigate if the growth pattern of CD children starts to deviate from the reference population already at birth (0 year), or at the time children commence to eat gluten (1/2 year) or later (at 1 year).

**Table 1:** Growth screening rules with their definitions, interpretation of the used parameters and cut off (simulation) values (see method for details)

Screening rule	Definition	Parameter	Interpretation	Simulation values
Delta rule*^^^	For ages *e*_1 _to 2.5 years, refer if	*e*_1_	Age (in years) after which the rule is effective	0, 0.5, 1
	(SDS_2 _– SDS_1_) <*g*_1_	*g*_1_	Change in SDS	-0.5,-1,-1.5,-2,-2.5,-3

Extended delta rule*	For ages *e*_2 _to 2.5 years, refer if	*e*_2_	Age (in years) after which the rule is effective	0, 0.5, 1
	SDS_2_<*f*_1_, AND	*f*_1_	SDS cut off level below which the SDS_2 _must lie	-1,-1.3, -1.5,-2, -2.5
	(SDS_2 _– SDS_1_) <*g*_2_	*g*_2_	Change in SDS	-0.5,-1,-1.5,-2,-2.5,-3

Slowed growth *	For ages *e*_3 _to 2.5 years, AND	*e*_3_	Age (in years) after which the rule is effective	0, 0.5, 1
	X_2 _– X_1 _≥ *3/12 *refer if		Minimal three months interval between ages X_1 _and X_2_	
	SDS_2_<*f*_2_, AND	*f*_2_	SDS cut off level below which the SDS_2 _must lie	-1,-1.3, -1.5,-2, -2.5
	(SDS_2_- SDS_1_)/(X_2_-X_1_) < g_3_	*g*_3_	Change in SDS per year	-0.5,-1,-1.5,-2,-2.5

Conditional weight gain rule	For ages *e*_4 _to 2.5 years, refer if	*e*_4_	Age (in years) after which the rule is effective	0, 0.5, 1
	weight SDS_2 _<*f*_3 _AND	*f*_3_	SDS cut off level below which SDS_2 _must lie	-1,-1.3, -1.5,-2, -2.5
	weight SDSgain = (weight SDS_2 _– r weight SDS_1_)/(√1-r^2^) <*g*_4_	*g*_4_	Change in SDS	-0.5,-1,-1.5,-2,-2.5

Absolute SDS rule*	For ages 0 to *e*_5 _years, refer if	*e*_5_	Age (in years) at which the referral level changes	0, 0.5, 1
	SDS <*f*_4_	*f*_4_	SDS cut off level before age *e*_5_	-1, -1.3, -1.5, -2, -2.5, -3, -3.5
	For ages *e*_5 _to 2.5 years, refer if SDS <*f*_5_	*f*_5_	SDS cut off level after age *e*_5_	-1, -1.3, -1.5, -2, -2.5, -3

Parental height corrected rule	For ages *e*_6 _to 2.5 years, refer if	*e*_6_	Age (in years) after which the rule is effective	0, 0.5, 1
	length SDS <*f*_6_, AND	*f*_6_	Length SDS must lie below this cut off level	-1, -1.3, -1.5, -2, -2.5
	length SDS – TH SDS <*g*_5_	*g*_5_	Difference between length SDS and target height (TH) SDS	-1, -1.3, -1.5, -2, -2.5

Parental height deflection rule	For ages *e*_7 _to 2.5 years, refer if	*e*_7_	Age (in years) after which the rule is effective	0, 0.5, 1
	(length SDS_2 _– length SDS_1_) <*g*_6_, AND | length SDS_2 _– TH SDS | > | length SDS_1 _– TH SDS |	*g*_6_	Change in length SDS Length SDS at age X_1 _is closer to it's target height than length SDS at age X_2_	-0.5,-1,-1.5,-2,-2.5,-3

Combined weight and length deflection rule	For ages *e*_8 _to 2.5 years, AND	*e*_8_	Age (in years) after which the rule is effective	0, 0.5, 1
	(weight SDS_2 _– weight SDS_1_) <*g*_7_, AND	*g*_7_	Weight change in SDS	-0.25,-0.5,-1,-1.5,-2
	(length SDS_2 _– length SDS_1_) <*g*_8_, AND Y_1 _> X_1_	*g*_8_	Length change in SDS Starting point length deflection (Y_1_) after starting point weight deflection (X_1_)	-0.25,-0.5,-1,-1.5,-2

Several screening rules for growth were studied. Each screening rule consists of parameters that we have varied. For more details, see the paragraph screening rules.

* Calculated for length (height), weight, and BMI

^^^For example, if *e*_1 _= 0.5 year and *g*_1 _= -2 weight SDS, then a child is referred if the second weight SDS measurement is -2 below the first weight SDS measurement and both weights were measured after six months of age (or at six months of age for the first measurement).

In total, we formulated eight rules, and each rule is explained in detail in Table 1 and below.

1. The first rule (*delta rule*) refers a child if an absolute change in length SDS, weight SDS or BMI SDS occurs. For example, suppose a child has two weight measurements, one measurement at the age of six months and one at the age of 1.5 years. This child will then be referred according to the delta rule with parameters *e*_1 _= 0.5 and *g*_1 _= -2 (see Table 1) if his or her weight decreases by more than 2 SDS between the first and the second measurement.

2. The second rule (*extended delta rule*) is equal to the first rule with the extension that the second measurement has to have a low SDS (for example less than -1.5 SDS).

3. The third rule (*slowed growth rule*) signals whether an abnormal slowed growth for length, weight or BMI occurs in terms of change in SDS per year in combination with a current low SDS. For example, suppose a child has two length measurements, one measurement at the age of seven months and one measurement six months later. This child will then be referred according to the slowed growth rule with parameters *e*_3 _= 0.5, *g*_3 _= -1 and *f*_2 _= -1.5 (see Table 1) if the difference between the second and first length measurement per year exceeds 1 SDS (which corresponds to a decrease of 0.5 SDS within six months) and if the second measurement is less than -1.5 SDS. We prefer the term *slowed *growth over the term *velocity *to indicate the decrease in growth in SDS per year. The term velocity commonly refers to cm or kg/year.

4. The fourth rule (*conditional weight gain rule) *is the conditional weight gain rule that signals whether a child's conditional weight gain SDS is less than a certain value [17,18] with the restriction of having a low weight SDS.

5. The fifth rule (*absolute SDS rule) *refers a child if the length SDS, weight SDS or BMI SDS is low. An example is to refer if a child's length SDS is less than -2 (*e*_5 _= 0 and *f*_5 _= -2).

6. We also considered rules that take genetic height potential into account. The sixth rule (*parental height corrected rule*) compares the height SDS of the child to its target height SDS in combination with a low height SDS.

7. The seventh rule (*parental height deflection rule*) signals whether a slowed growth for length SDS of the child moves away from the child's target height. This rule was added because of the assumption that a correction might be needed for parental height in the first years of life: e.g. a baby that is born with a length SDS of -1 and has a target height SDS of +2, would be expected to cross the SD lines in upward direction in the first 2–3 years. A growth disorder could disturb this, and a stable length SDS of this child at -1 over the first 2 years could indicate growth pathology such as CD.

8. Similarly, in the eight rule (*combined weight and length deflection rule) *we combined weight and length, in which a slowed growth for length occurs after a slowed growth for weight.

Several cut off values for age were used as the effectiveness of these rules may increase by examining higher age groups. Slowed growth requires measurements taken at least three months apart. We chose this short time interval to facilitate early detection, taking into consideration that children in the first year of life grow faster than in later years.

It should be noted that some parameters select a subset of the data and assume multiple measurements. The rules were only tested on children that complied with these assumptions. All available pairs of measurements for each infant were used.

### Statistical analysis

Each screening rule was implemented using S-Plus version 7.0.3 for Microsoft Windows (2005), and was applied to the longitudinal data of children. We calculated sensitivity, specificity and positive predictive value (PPV) for each rule with several scenarios (simulation values). The rules were ordered according to their sensitivity at high levels of specificity. A higher sensitivity at the same level of specificity, results in a better performance. The results were plotted as a Receiver Operating Characteristic (ROC) curve, but scaled to a different axis than conventionally in order to view the area of most interest (high specificity). Each point in the ROC curve is the false-positive rate against sensitivity of a scenario (combination of simulation values) of a rule. Scenarios of rules with approximately 2% false-positive rates were presented in detail as we assumed that a false-positive rate greater than 2% would result in too many referrals. PPV was calculated assuming that the incidence of CD is 0.54 per 1000 live births in the Caucasian population [9]. Sensitivity analyses were performed to calculate the effect of small variations (0.1–1.0/1000) in the incidence of CD on PPV.

Length, weight and BMI were expressed as SDS, using the Dutch reference growth data [19,20]. In preterm infants (gestational age < 37 weeks) length and weight SDS were corrected for gestational age. The intrauterine growth charts from the Swedish reference population was used to express SDS up to the age corresponding with 40 weeks of gestation [21]. Between 40 and 42 weeks an interpolation between the growth curve of the Swedish reference population and that of the Dutch reference population was used. From 42 weeks of gestation till the age of 2 years, SDS was calculated on ages corrected for gestational age, using the Dutch reference growth data.

We assumed that a child was referred if the growth pattern met the criteria of a given screening rule for the first time. All rules were dealt with separately, meaning that the same child could be referred according to each separate rule.

Written informed consent was obtained from the patient for publication of this case report and accompanying images. A copy of the written consent is available for review by the Editor-in-Chief of this journal.

## Results

Table 2 contains general characteristics of the symptomatic CD group and the screened CD group. In the symptomatic group, mean weight SDS was compromised most, followed by mean BMI SDS.

**Table 2:** General characteristics of the CD-population

Characteristic	Screened (n = 26)	Symptomatic (n = 96)
Gender (M)	50%	35%
Ethnicity		
Dutch	92%	98%
Others	8%	2%
Median (range) age in years at start diet	3.96 (2.94–6.06)	1.43 (0.41–20.7)
Mean (SD) length SDS *∞	-0.26 (0.98)	-0.89 (1.30)
Mean (SD) weight SDS*∞	-0.06 (0.81)	-1.54 (1.15)
Mean (SD) BMI SDS*∞	0.28 (0.57)	-1.28 (1.15)
Mean (SD) target height SDS	0.41 (0.92)	0.00 (0.75)

* For the children in the screened group figures at diagnosis are given (also when diagnosis is after 2.5 years of age). For the symptomatic CD children figures at the start of the gluten-free diet are given.

∞ Based on children with at least one measurement between 6 months before and 3 months after gluten-free diet or diagnosis.

### Diagnostic performance of the rules: screened CD children

All screening rules detected less than 5% of the screened CD children at a 2% false-positive rate. Therefore, none of the rules were able to discriminate between the CD children detected by screening and the reference children. This indicates that the screened and the reference children hardly differ in terms of their growth pattern.

### Diagnostic performance of the rules: symptomatic CD children

**Figure 2:**
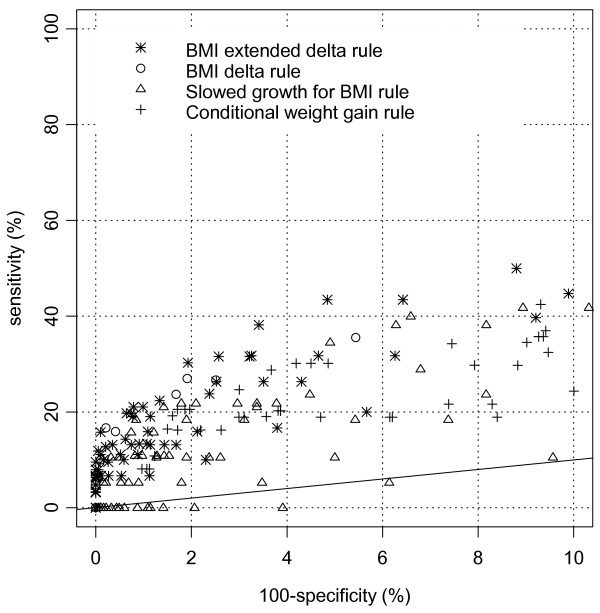
**ROC plot of effective growth screening rules for detecting CD in the symptomatic group**. The rules are an absolute change in BMI SDS with or without the restriction of a low BMI SDS, a slowed growth for BMI, and a conditional weight gain in combination with a low weight SDS.

The results are different for the symptomatic CD children. Figure 2 shows the ROC plot for the four best screening rules for the symptomatic CD group. Only scenarios with a false-positive rate of less than 10% are plotted. The line for which sensitivity is equal to 100-specificity is given in the figure. Scenarios on this line are not able to discriminate between the CD and the reference group. The BMI extended delta rule had the highest sensitivities at low false-positive rates. A strict version of this rule is a decrease in BMI SDS of -3 and a BMI SDS less than -1 between birth and 2.5 years of age. This scenario correctly identified 21% (95%-CI 12–30) of the CD children and 99% (95%-CI 98.6–99.4) of the reference children were correctly labelled as disease free. The PPV of this scenario is approximately 1%. For example, suppose a boy has a BMI on the line above the median of the growth chart (SDS = +1) at three months of age. If he crosses three SDS lines (SDS 0, -1 and -2) before the age of 2.5 years, then the boy has a 1% probability of having CD. A less strict version of the BMI extended delta rule is a decrease in BMI SDS of at least -2 and a BMI SDS of less than -1.5 between birth and 2.5 years of age, with a sensitivity of 38% (95%-CI 27–49), a false-positive rate of 3.4% (95%-CI 2.6–4.2) and a PPV of 0.60%.

The properties of the four best rules for the symptomatic CD group, in terms of sensitivity and PPV at approximately 98% specificity, are presented in table 3. Thirty percent (95%-CI 20–40) of the CD children and 1.9% (95%-CI 1.3–2.5) of the reference children had a decrease in BMI SDS of at least -2.5 and a BMI SDS less than -1.5 between birth and 2.5 years of age. In children with such decrease in BMI SDS, the probability of CD is 0.85%. PPV varied between 0.16% and 1.57% when changing the incidence of CD from 1:10000 to 1:1000 live births. For example, a girl has a BMI on the median of the growth chart at one month of age, and her BMI crosses centiles for a certain time period until she reaches a BMI SDS of less than -2.5. Then this girl will be referred according to the scenario above. Her probability of actually having CD is 0.85%. Furthermore, 27% (95%-CI 16–38) of the CD children versus 1.9% (95%-CI 1.3–2.5) of the reference children had a decrease in BMI SDS of at least -1.5 when they were older than six months of age. The probability of having CD when a child complies with this rule is 0.76%. Both the slowed growth for BMI rule and the conditional weight gain rule result in a sensitivity of approximately 22% (95%-CI 11–33) at a false-positive rate of 1.9% (95%-CI 1.3–2.5). The PPV is approximately 0.6%. The sensitivity between the first rule (the BMI extended delta rule) and the fourth rule (conditional weight gain rule) differed most. Twenty percent of the CD children complied with the first rule (true-positive) and not with the fourth rule (false-negative), while ten percent of the CD children complied with the fourth rule and not with the first rule. If we combine both rules, sensitivity is 41%. However, the false-positive rate also increased to 3.6%.

**Table 3:** Simulation values and the percentage of detected CD children (sensitivity) with approximately 2% false-positives (= 98% specificity)

(symptomatic) CD	Simulation values*			Sensitivity (95%-CI)	100-Specificity (95%-CI)	PPV
BMI extended delta rule	*e*_2 _= 0	*f*_1 _= -1.5	*g*_2 _= -2.5	30 (20–40)	1.9 (1.3–2.5)	0.85%
BMI delta rule	*e*_1 _= 0.5		*g*_1 _= -1.5	27 (16–38)	1.9 (1.3–2.5)	0.76%
Slowed growth for BMI rule	*e*_3 _= 0	*f*_2 _= -2	*g*_3 _= -2.5	22 (11–33)	1.8 (1.2–2.4)	0.65%
Conditional weight gain rule	*e*_4 _= 0.5	*f*_3 _= -2.5	*g*_4 _= -0.5 to -1.5	21 (12–30)	1.9 (1.3–2.5)	0.58%

*Table 1 explains the interpretation of the simulation values

The delta rules for length and weight, the slowed growth rule for length and weight, the absolute SDS rule, rules that take genetic height potential into account (parental height corrected rule and parental height deflection rule) and the combined weight and length deflection rule proved less effective (data not shown). At a fixed specificity of 98%, sensitivities for these rules were less than 20%.

## Discussion

Our study shows that for detecting or predicting symptomatic CD children by growth, a decrease in BMI is more informative than a decrease in weight or length. The screened CD children grow normally between birth and 2.5 years of age.

The optimal weight rule in this study was the conditional weight gain rule. The conditional weight gain rule corrects for regression to the mean. The amount of regression to the mean depends on the correlation of body weight across age [17]. The correlations that we used in our study were based on children in the UK [18]. The conditional weight gain rule may perform better when using correlations of Dutch children. However, these correlations are presently not available. To validate the UK correlations for the Dutch children, we calculated if the SDS_gain _has a mean of zero and a SD of 1, and if it is uncorrelated with the first weight SDS. For the reference group of Dutch children, the mean (SD) SDS_gain _is -0.06 (1.41) and its correlation with the first weight SDS is -0.23. As both SD and correlation are quite high, the conditional weight gain rule may perform better when using Dutch correlations of weights. Furthermore, a rule based on BMI that corrects for regression to the mean may improve discrimination between the symptomatic CD group and the reference group. So far no suitable correlations have been published to calculate this conditional gain.

In this study, PPV of the screening rules may be underestimated for several reasons. Firstly, PPV will be slightly higher as there will be one case of CD in our reference group if we assume that the incidence of CD is 0.54 per 1000 life births. Secondly, PPV was calculated using only the incidence of CD. However, if we keep in mind that children with genetic disorders or diseases other than CD may be detected by some of our rules for failure to thrive, PPV may be higher. For example, if we assume that sensitivity and specificity for the most optimal rule for CD in this study is similar to patients with Cystic Fibrosis (CF), then PPV will be higher if this is based on the incidence of both CD and CF [22].

As Csizmadia et al. reported earlier, the children with CD detected by screening had a normal weight and length at time of diagnosis [9]. We have confirmed that all children in this group indeed had a normal growth pattern between birth and 2.5 years of age. This corresponds with the asymptomatic character of this silent form of CD. Thus monitoring growth would not seem to be useful for the detection of silent CD at this specific stage of the disease. The prevalence of children with short stature and no gastrointestinal symptoms investigated for CD is 2–8% [7] compared to a prevalence of 1:300 to 1:100 in the general population. Therefore, one may expect that these children would develop abnormal growth after several years.

CD is often atypical or clinically silent, which results in many undiagnosed children. However, since the widespread introduction of serologic testing and the increased awareness of CD in the late 1990s there has been an increase in incidence as well as a change in clinical presentation [3-6]. The classical symptoms, such as malabsorption and poor weight gain no longer dominate the clinical picture. Instead, there is an increase of cases with non-classical symptoms, including unusual intestinal complaints or extra-intestinal symptoms (e.g. short stature) involving older children. In addition, the age of presentation may be changing due to differences in infant feeding practices, duration of breastfeeding and improved recognition of potential CD by general practitioners. As our non-screened population was diagnosed before 1995, we were not able to study the effect of this change in time on the performance of the growth criteria. However, one may assume that for the age group included in our study, the performance of the growth criteria is similar for the present CD-population, as it is mainly the delayed onset variant of the disease (the non-classical form) that has increased during the recent years, suggesting that the growth impairment becomes apparent much later.

Most of the patients in our study were females, as was reported in several other studies [23]. Bardella et al. hypothesized that males escape diagnosis, but that the two sexes are equally affected. This hypothesis is supported by the absence of differences in sex in the screening study (see Table 2).

In conclusion, we support the clinician to consider testing for CD in a diagnostic work-up in young children with failure to thrive. The most sensitive growth parameter is BMI SDS. We recommend further research with a large sample of children with CD diagnosed in the last few years to study the most valid simulation values for referral rules based on BMI and other diagnostics.

## Conclusion

BMI is more efficacious than weight, and much more than length or height, in detecting symptomatic children with CD. Toddlers with CD detected by screening grow normally at this stage of the disease.

## Competing interests

The authors declare that they have no competing interests.

## Authors' contributions

PvD performed the statistical analyses and wrote the methods, results and part of the discussion paragraphs. FKG wrote all other paragraphs. SMPFdMKS was involved in all versions of the manuscript. FKG, BB, GMD and CGC were responsible for the collection of the data. JMW, WO, SvB and PHV initiated the study and were involved in all versions of the manuscript.

## Pre-publication history

The pre-publication history for this paper can be accessed here:


